# Baicalein protects against oxLDL-caused oxidative stress and inflammation by modulation of AMPK- alpha

**DOI:** 10.18632/oncotarget.12788

**Published:** 2016-10-20

**Authors:** Kun-Ling Tsai, Ching-Hsia Hung, Shih-Hung Chan, Jhih-Yuan Shih, Yung-Hsin Cheng, Yi-Ju Tsai, Huei-Chen Lin, Pei-Ming Chu

**Affiliations:** ^1^ Department of Physical Therapy, College of Medicine, National Cheng Kung University, Tainan, Taiwan; ^2^ Institute of Allied Health Sciences, College of Medicine, National Cheng Kung University, Tainan, Taiwan; ^3^ Department of Internal Medicine, National Cheng Kung University Hospital, College of Medicine, National Cheng Kung University, Tainan, Taiwan; ^4^ Department of Internal Medicine, Chi-Mei Hospital, Tainan, Taiwan; ^5^ Department of Education and Research, Taipei City Hospital, Taipei, Taiwan; ^6^ Department of Physical Therapy, Shu-Zen Junior College Of Medicine And Management, Taiwan; ^7^ Department of Anatomy, School of Medicine, China Medical University, Taichung, Taiwan

**Keywords:** atherosclerosis, ROS, endothelial cells, lectin-like oxidized LDL receptor 1, baicalein, Gerotarget

## Abstract

Atherosclerosis is considered to be a form of chronic inflammation and a disorder of lipid metabolism. Oxidative transformations in the lipid and apolipoprotein B (Apo B) constituent of low density lipoprotein drive the initial step in atherogenesis due to macrophage scavenger receptors identify oxidized LDL (oxLDL) but non-oxidized LDL. The human vascular endothelial cells fact a critical role in vasodilation, provides a nonadhesive surface for circulation, reduces vascular smooth muscle proliferation, inflammation, thrombus formation and platelet aggregation. Assembly of oxLDL contribute to stimulation of endothelial cells with up-regulation of adhesion molecules, increase oxidative stress to the vascular endothelium and inhibition of NO-mediated vasodilation. When adhesion molecules are over-expressed on the surface of endothelial cells under oxLDL stimulation, it will recruit monocytes to the arterial wall. Then adherent monocytes will migrate into the subendothelial space and subsequently differentiate into macrophages. In the subendothelial space, oxLDL will be taken up by macrophages, thereby causing the substantial cholesterol accumulation and the foam cells production.

## INTRODUCTION

Atherosclerosis is considered to be a form of chronic inflammation and a disorder of lipid metabolism [1]. Oxidative transformations in the lipid and apolipoprotein B (Apo B) constituent of low density lipoprotein drive the initial step in atherogenesis due to macrophage scavenger receptors identify oxidized LDL (oxLDL) but non-oxidized LDL [2]. The human vascular endothelial cells act a critical role in vasodilation, provides a nonadhesive surface for circulation, reduces vascular smooth muscle proliferation, inflammation, thrombus formation and platelet aggregation [3]. Assembly of oxLDL contribute to stimulation of endothelial cells with up-regulation of adhesion molecules, increase oxidative stress to the vascular endothelium and inhibition of NO-mediated vasodilation [4]. When adhesion molecules are over-expressed on the surface of endothelial cells under oxLDL stimulation, it will recruit monocytes to the arterial wall. Then adherent monocytes will migrate into the subendothelial space and subsequently differentiate into macrophages [5]. In the subendothelial space, oxLDL will be taken up by macrophages, thereby causing the substantial cholesterol accumulation and the foam cells production [6].

Lectin-like Oxidized LDL receptor 1 (LOX-1), also known as scavenger receptor class E member 1 (SR-E1). It is expressed in vascular smooth muscle cells, macrophages and vascular endothelial cells [7, 8]. Alike to other type of scavenger receptors, LOX-1 binds various ligands. Such as polyinosinic acid and phosphatidylserine [9], activated platelets [10]. LOX-1 has been revealed with the ability to bind oxLDL, but not native LDL [11]. A previous study revealed that oxLDL led to LOX-1 over-expression also activated NADPH oxidase (both gp91phox and p47phox subunits) expression and enhanced ROS generation in human coronary artery endothelial cells stimulated with. They proposed the evidence for the pathway use of anti-LOX-1 antibody markedly reduced oxLDL-induced ROS generation and NADPH oxidase expression [12].

The AMP-activated protein kinase (AMPK) is one heterotrimeric serine/threonine protein kinase. AMPK is up-activated in many varied cell types by enhancing intracellular concentrations of AMP [13]. Several studies suggested that AMP-activated protein kinase (AMPK) is not only in controlling metabolic homeostasis but also in cardiovascular pathology. Moreover, Wang et al showed that AMPKα deficient mice increased the expression of p47phox, p67phox, and gp91phox compared to their wild type counterparts leading to enriched ROS generation and accelerated degradation of I-κB, resulting in excessive up-regulation of NF-κB and consequent NADPH oxidase activation [14]. They revealed that AMPKα functions as a physiological inhibitor of NADPH oxidase and ROS production in endothelial cells. Ceolotto et al also showed that endothelial cells incubated with a pharmacological activator of AMPK (AICAR) protects against high glucose-caused NADPH oxidase over-expression and ROS formation by PKC inhibition [15]. Therefore, AMPK maintains the anti-atherogenic and anti-inflammatory phenotype of human endothelial cells.

Baicalein is one natural phenolic antioxidant which is isolated from Scutellaria baicalensis (*S. baicalensis*) Georgi (Huangqinin Chinese). *S. baicalensis Georgi* is one extensively used herb employed in China and also in other countries. In traditional Chinese medicines, extracts from the *Scutellaria radix* have been applied for anti-cancer, anti-inflammation, repressing the cholesterol concentrations and repressing blood pressures [16, 17]. In this study, we aimed to explore whether Baicalein could mitigate LOX-1-drived human endothelial dysfunction by the up-regulation of NADPH oxidase, and if so, whether AMPK was involved in the process.

## RESULTS

### Effect of baicalein on LOX-1 protein expression induced by oxLDL

**Figure 1:**
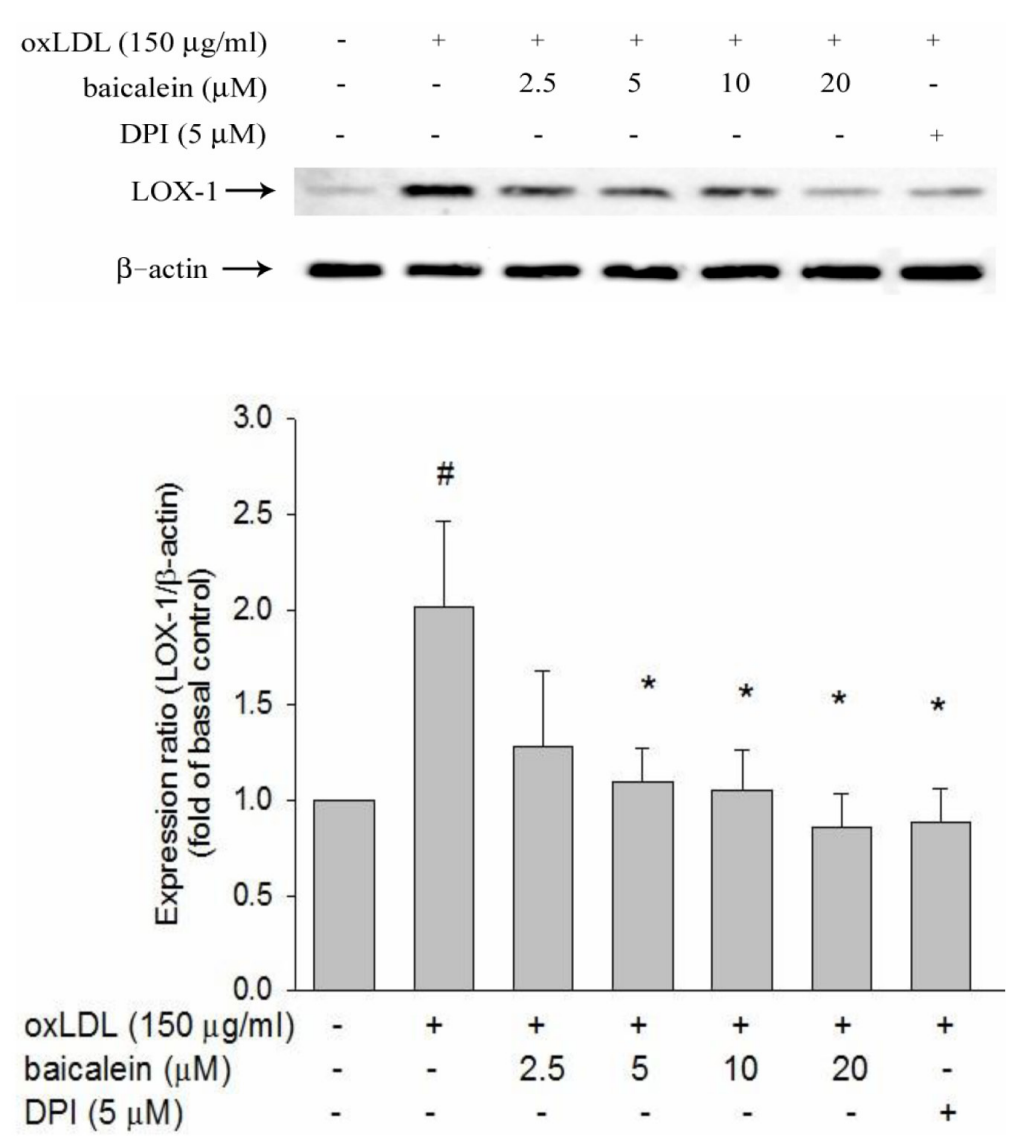
Inhibitory effects of baicalein on oxLDL-induced endothelial LOX-1(a lectin-like oxLDL receptor-1) protein expression The expression of protein were analyzed by Western blot respectively pretreated with baicalein (2.5-20 μM) or DPI (5 μM) for 2 hrs followed by exposure to oxLDL (150 μg/ml) for a further 24 hrs period in HUVECs. The levels of LOX-1 protein were normalized to the level of β-actin. Data of bar figure represent mean±SEM of 3 independent analyses. # *P* < 0.05 compared with control and *P < 0.05 compared with oxLDL-stimulated HUVECs.

LOX-1 binds structurally diverse ligands and oxLDL has been identified with the ability to bind LOX-1 [11]. The expression levels of LOX-1 protein expression in HUVECs were enhanced by oxLDL (150 μg/ml). Treatment of HUVECs with baicalein for 2 hrs at concentrations above 2.5 μM before exposure to oxLDL for 24 hrs resulted in suppression of LOX-1 expression both in protein levels (Figure 1). In addition, pretreatment with a free radical inhibitor (DPI) significantly mitigates oxLDL-facilitated LOX-1 up-regulation.

### Effects of baicalein on oxLDL-induced NADPH oxidase activation

**Figure 2:**
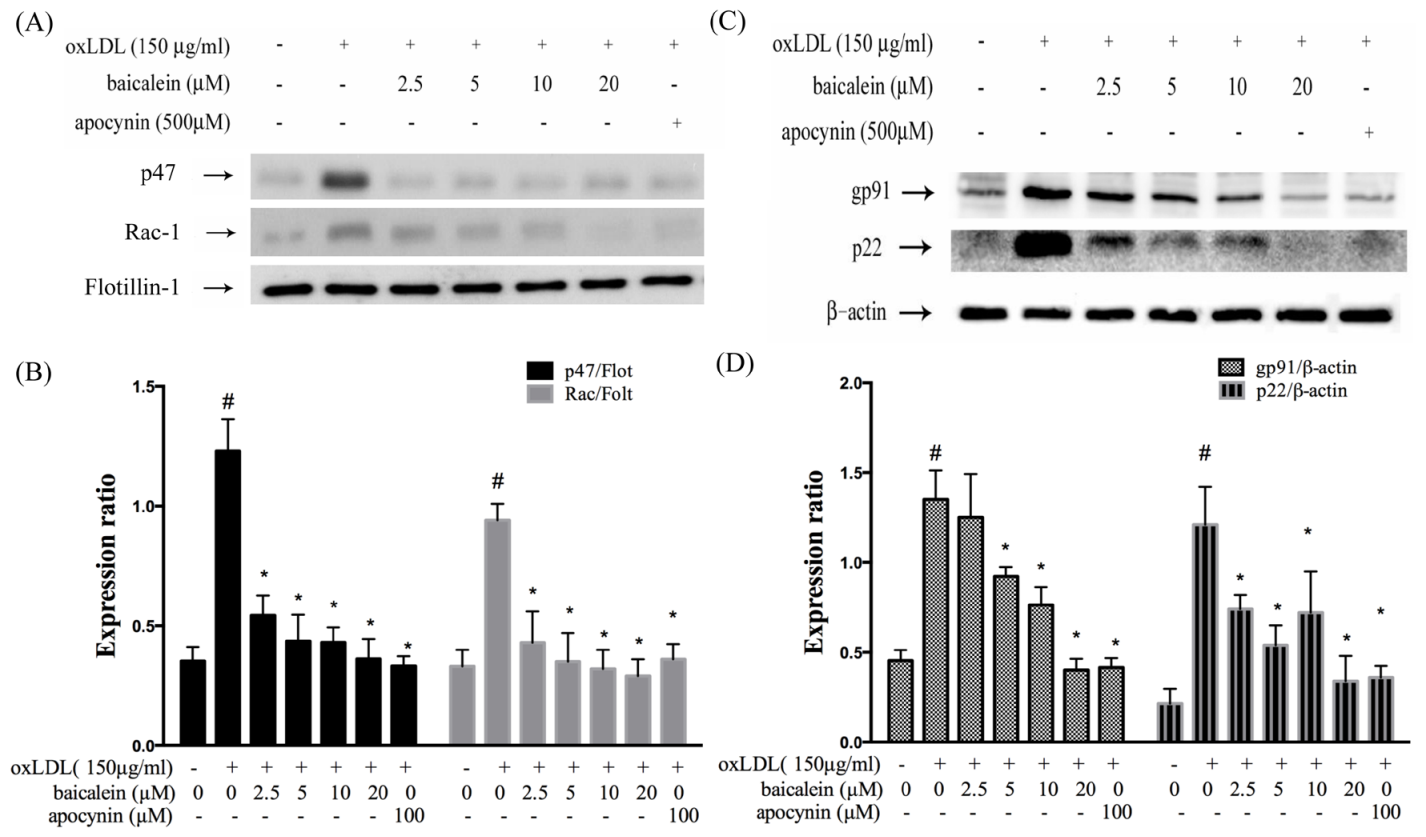
Baicalein attenuated the level of NADPH oxidase membrane assembly HUVECs were pretreated for 2 hrs with the indicated concentrations of baicalein followed by stimulation with oxLDL (150 μg/mL) for 24 hrs. Representative Western blots **A.**, **C.** and summary data **B.**, **D.** showed that baicalein protected against oxLDL-induced p47phox and Rac-1 translocation to the plasma membrane, and gp91 as well as p22phox expression. The levels of cytosolic protein and membrane protein were normalized to the levels of β-actin and flotillin-1, respectively. Data are mean±SEM of three different experiments. # *P* < 0.05 compared with control and *P < 0.05 compared with oxLDL-stimulated HUVECs.

Previous study has reported a predominant function of vascular NADPH oxidases in free radical formation. The effects of baicalein on gp91phox and p22phox expression were examined by western blotting analysis in HUVECs and membrane translocation of p47phox and Rac-1 were investigated in HUVECs with membranes and cytosolic isolation. We found that the expression of p47phox and Rac-1 in membrane fractions were 2~3-fold higher in HUVECs stimulated with oxLDL for 1hr than those in control HUVECs (Figure 2A, 2B). The enhanced p47phox and Rac-1 on membrane translocation by oxLDL were inhibited by pretreatment with baicalein (20 μM). Furthermore, we also confirmed that the total protein expression of gp91phox and p22phox were increased in endothelial cells stimulated to oxLDL for 24 hrs. As expected, pretreatment of oxLDL-stimulated HUVECs with baicalein (2.5-20 μM) led to a reduction in gp91phox and p22phox protein expression. As well as, they were inhibited by NADPH oxidase inhibitor apocynin (Figure 2C, 2D).

### ROS generation and antioxidant enzyme expression

**Figure 3:**
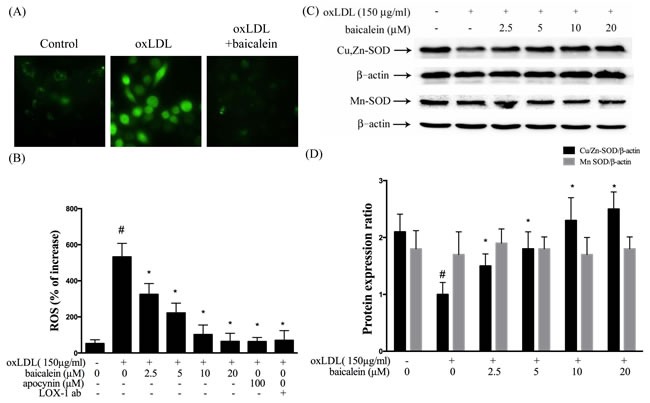
Inhibitory effects of baicalein on oxLDL-induced ROS generation in HUVECs HUVECs were pretreated with baicalein (2.5-20 μM) or apocynin (100 μM) or LOX-1 antibody for 2 hrs followed by 1 hr incubation with fluorescent probe DCF-AM (10 μM), oxLDL(150 μg/ml) was then added to medium for 2 hrs. **A.** Fluorescence images show the ROS level in control cells (left) and HUVECs stimulated with oxLDL alone (middle) and in the presence of 20 μM baicalein (right). **B.** Fluorescence intensity of cells was measured with a fluorescence microplate reader. **C.**, **D.** Rrepresentative of Western blot of Cu, Zn-SOD and Mn-SOD protein levels in HUVECs pretreated with baicalein (2.5-20 μM) for 2 hrs, followed by 150 μg/ml oxLDL for 24 hrs. Data are mean±SEM of three different experiments. # *P* < 0.05 compared with control and *P < 0.05 compared with oxLDL-stimulated HUVECs.

Several lines of evidence implicate oxLDL increased production of ROS impairs endothelial function in humans, which subsequently cause to the endothelial dysfunction [18]. Therefore, we examined the influences of baicalein on the ROS formation (Figure 3A, 3B). The ROS concentrations significantly decreased in HUVECs which intervened with baicalein (1.25-20 μM) and apocynin (100 μM) for 2 hrs followed by exposure to oxLDL (150 μg/ml) in a dose-dependent manner. In addition, LOX-1 antibody (LOX-1 ab) also inhibited oxLDL-caused ROS formation.

Intracellular ROS are known to repress anti-oxidative enzymes. In Figure 3C and 3D, our data showed the protein levels of SOD-1 were suppressed by oxLDL stimulation and could be restored by baicalein treatment. However, oxLDL did not repress SOD-2 levels in endothelial cells.

### Effects of baicalein on oxLDL-induced protein kinase C and AMPK phosphorylation

**Figure 4:**
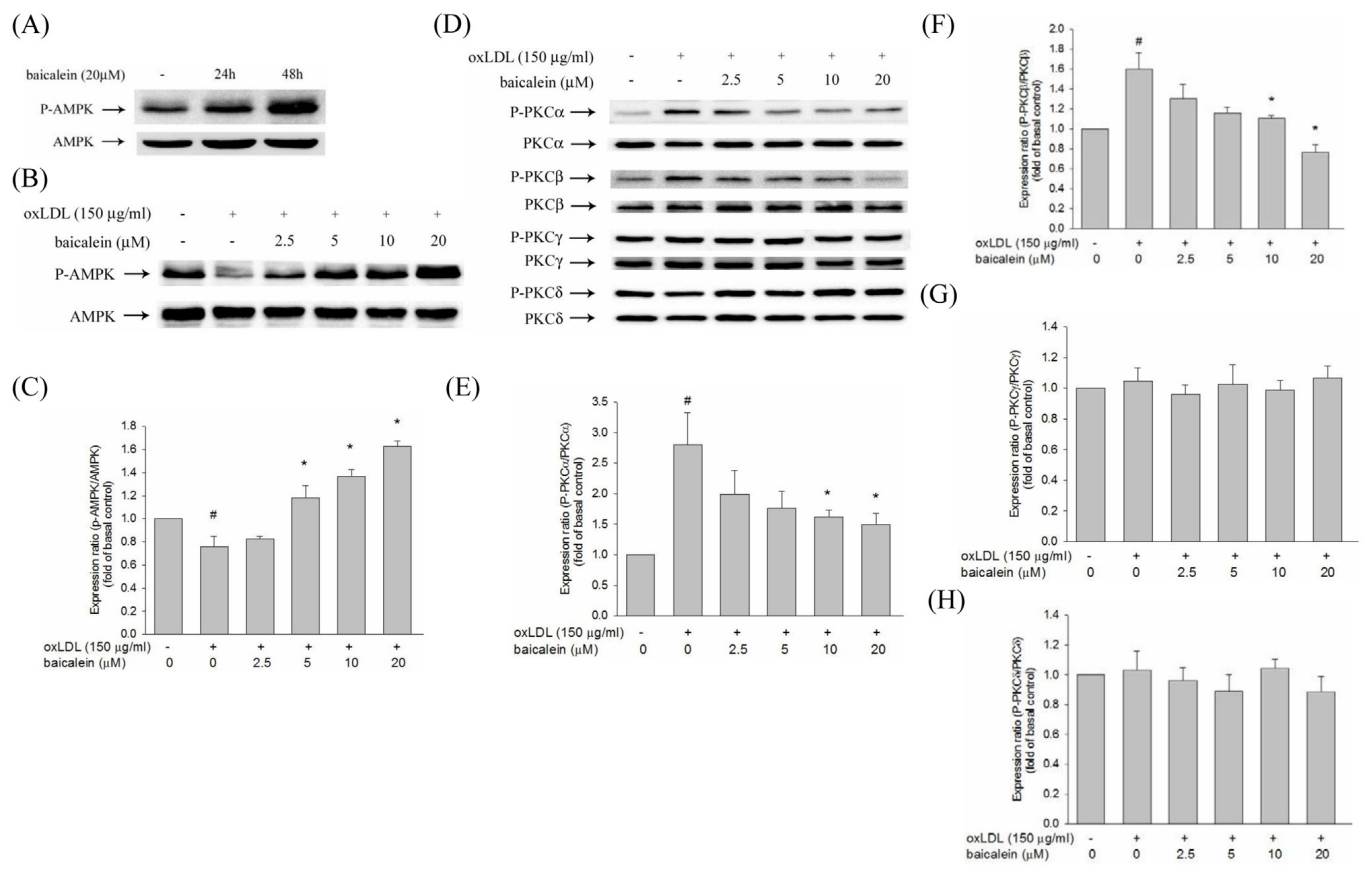
Effects of baicalein on oxLDL-impaired phosphorylation of AMPK and PKC activation in HUVECs The level of AMPK phosphorylation in HUVECs were treated with baicalein (20 μM) at 24 hrs and 48 hrs **A.** The protein expression of dephosphorylated and total AMPK were analyzed by Western blot pretreated with baicalein (2.5-20 μM) for 2 hrs prior to exposure to oxLDL(150 μg/ml) in HUVECs. AMPK **B.**, **C.** and PKC isoforms **D.**, **E.**, **F.**, **G.**, **H.** were analyzed by Western blot. Data of bar figure represent means±SEM of 3 independent analyses. # *P* < 0.05 compared with control and *P < 0.05 compared with oxLDL-stimulated cells.

**Figure 5:**
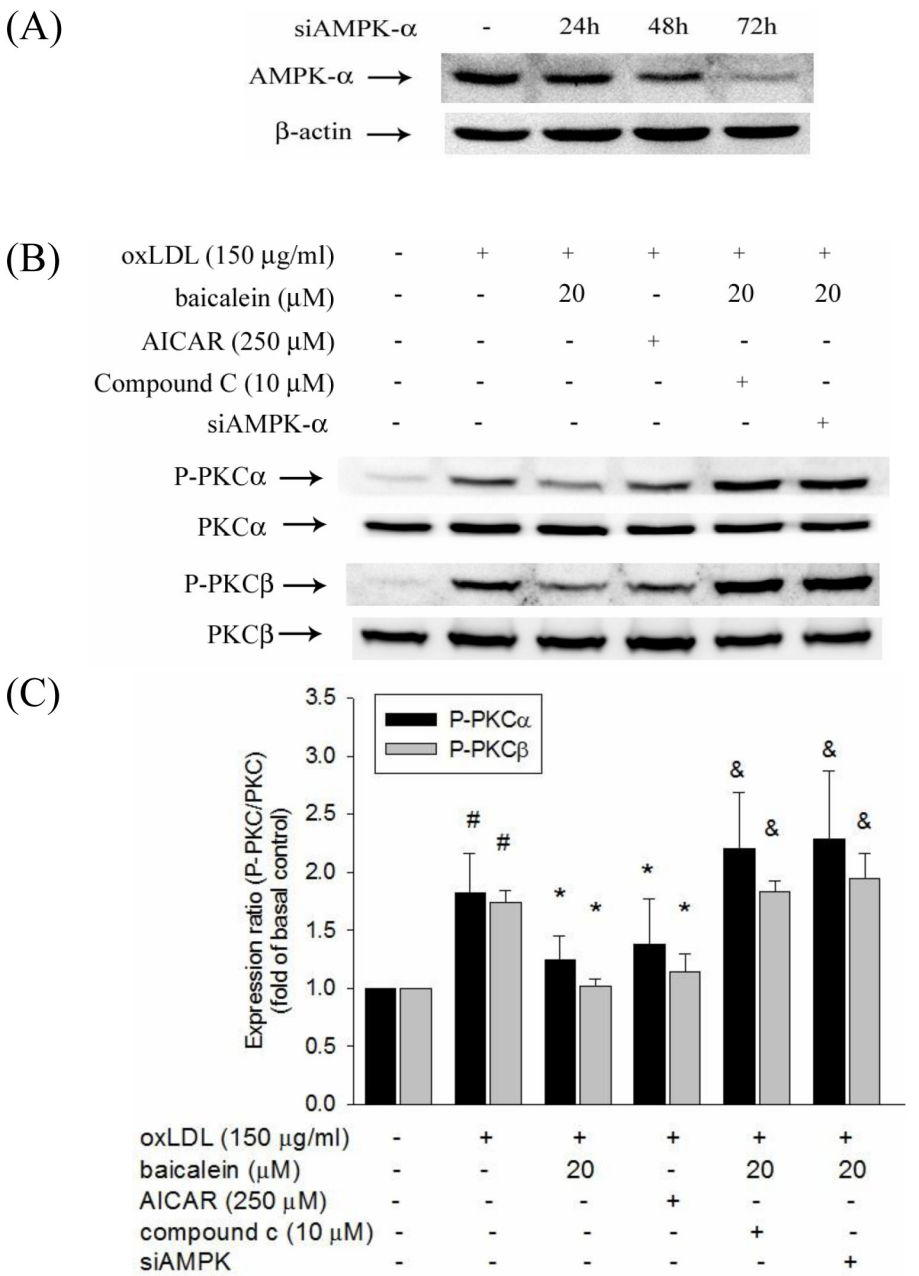
Effects of baicalein on oxLDL-induced phosphorylation of PKCα and PKCβ HUVECs were transfected with siAMPK-α or control scrambled siRNA. AMPK-α expression in siAMPK-α transfected cells at 24 hrs, 48 hrs, and 72 hrs **A.** The protein expression of phosphorylated and total PKCα and PKCβ were analyzed by Western blot pretreated with baicalein (20 μM) or AICAR (250 μM) for 2 hrs and compound c (10 μM) with baicalein (20 μM) prior to exposure to oxLDL(150 μg/ml) for 1 hr in HUVECs or pretreated with baicalein (20 μM) in siAMPK-transfected HUVECs. #*P* < 0.05 compared with control, & *P* < 0.05 compared with baicalein and *P < 0.05 compared with oxLDL-stimulated HUVECs.

There is growing evidence indicated that oxLDL-induced PKC has been shown to contribute to the up-regulation of NADPH oxidase as well as is dependent on AMPK regulation. We, therefore, investigate the effect of baicalein on AMPK and PKC activation to HUVECs induced by oxLDL. Our results showed that baicalein significantly increases AMPK phosphorylation in HUVECs (Figure 4A) and intervention of oxLDL-stimulated HUVECs with baicalein (2.5-20 μM) cause to significantly increase AMPK phosphorylation (Figure 4B, 4C). Stimulations of endothelial cells with oxLDL caused phosphorylation of protein kinase C α and β but not protein kinase γ and δ within 1 hr without affecting their protein levels (Figure 4D, 4E, 4F, 4G, 4H). Pretreatment with baicalein or AICAR reversed the oxLDL-induced phosphorylation of PKC α and β. Furthermore, this increase in PKC phosphorylation was not seen in cell pretreated with Compound C (an inhibitor of AMPK) or AMPK knockdown (Figure 5).

### Baicalein protects against oxLDL-caused NADPH oxidase activation by modulation of AMPK and PKC

**Figure 6:**
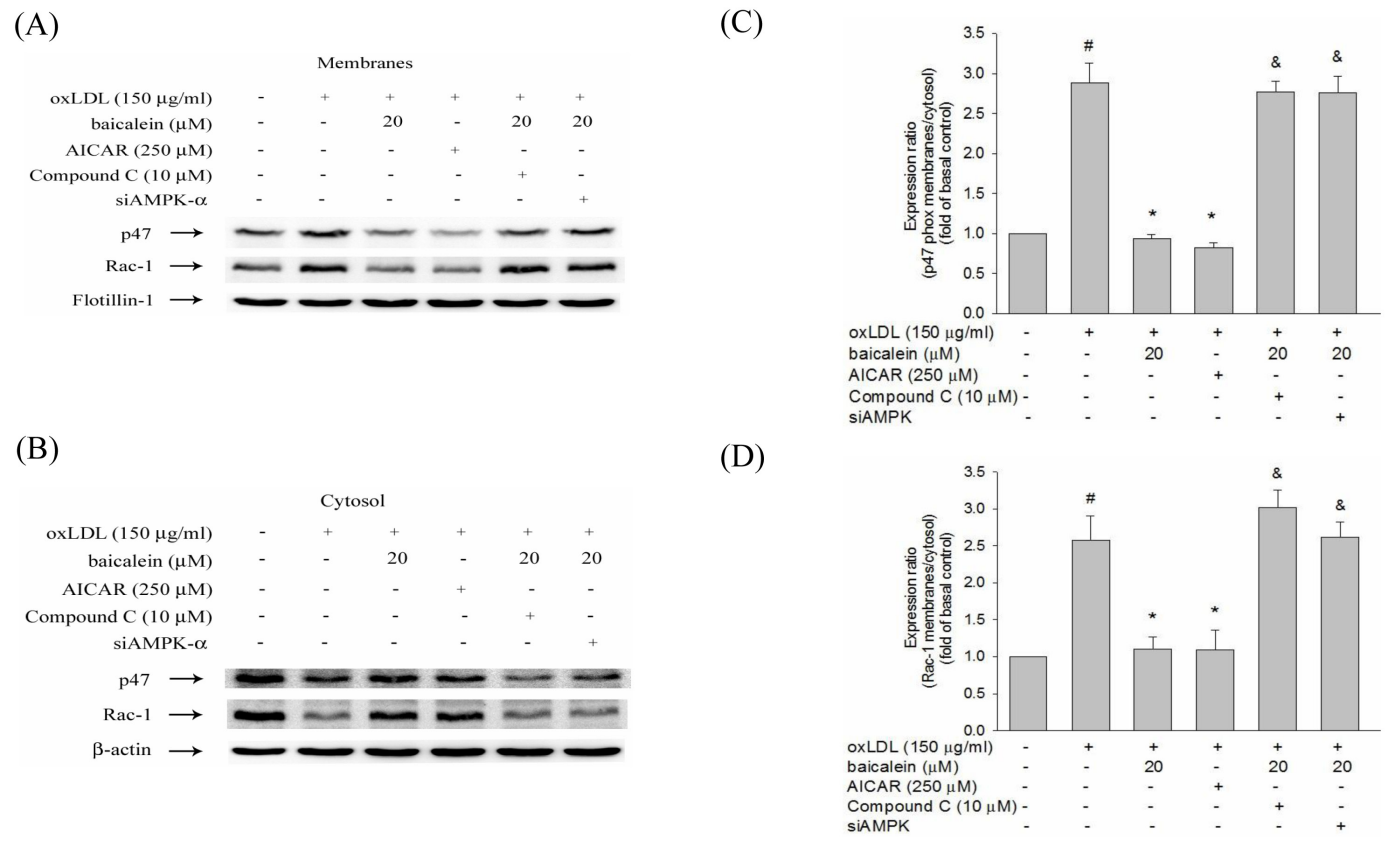
Effects of baicalein on oxLDL-induced subunit of NADPH oxidase complex p47phox and Rac-1 membrane translocation The protein expression of p47phox and Rac-1 were analyzed by Western blot pretreated with baicalein (20 μM) or AICAR (250 μM) for 2 hrs and Compound C (10 μM) with baicalein (20 μM) prior to exposure to oxLDL (150 μg/ml) for 1 hr in HUVECs or pretreated with baicalein (20 μM) in siAMPK-transfected HUVECs. Anti-flotillin-1 and anti-β-actin antibody were used for normalization of membranes and cytosolic proteins, respectively. Data of bar figure represent means±SEM of 3 independent analyses. # *P* < 0.05 compared with control, & *P* < 0.05 compared with baicalein 20 μM and *P < 0.05 compared with oxLDL-stimulated HUVECs.

In Figure 6, we found the expression levels of p47phox and Rac-1 in membrane fractions of endothelial cells were higher in HUVECs stimulated with oxLDL for 1hr than those in untreated HUVECs. Pretreatment with baicalein or AICAR mitigated the membrane shuttling of p47phox and Rac-1. Furthermore, this decrease in p47phox and Rac-1 membrane translocation was not seen in cell pretreated with Compound C or AMPK knockdown (Figure 6).

### Baicalein inhibits oxLDL-induced inflammation

**Figure 7:**
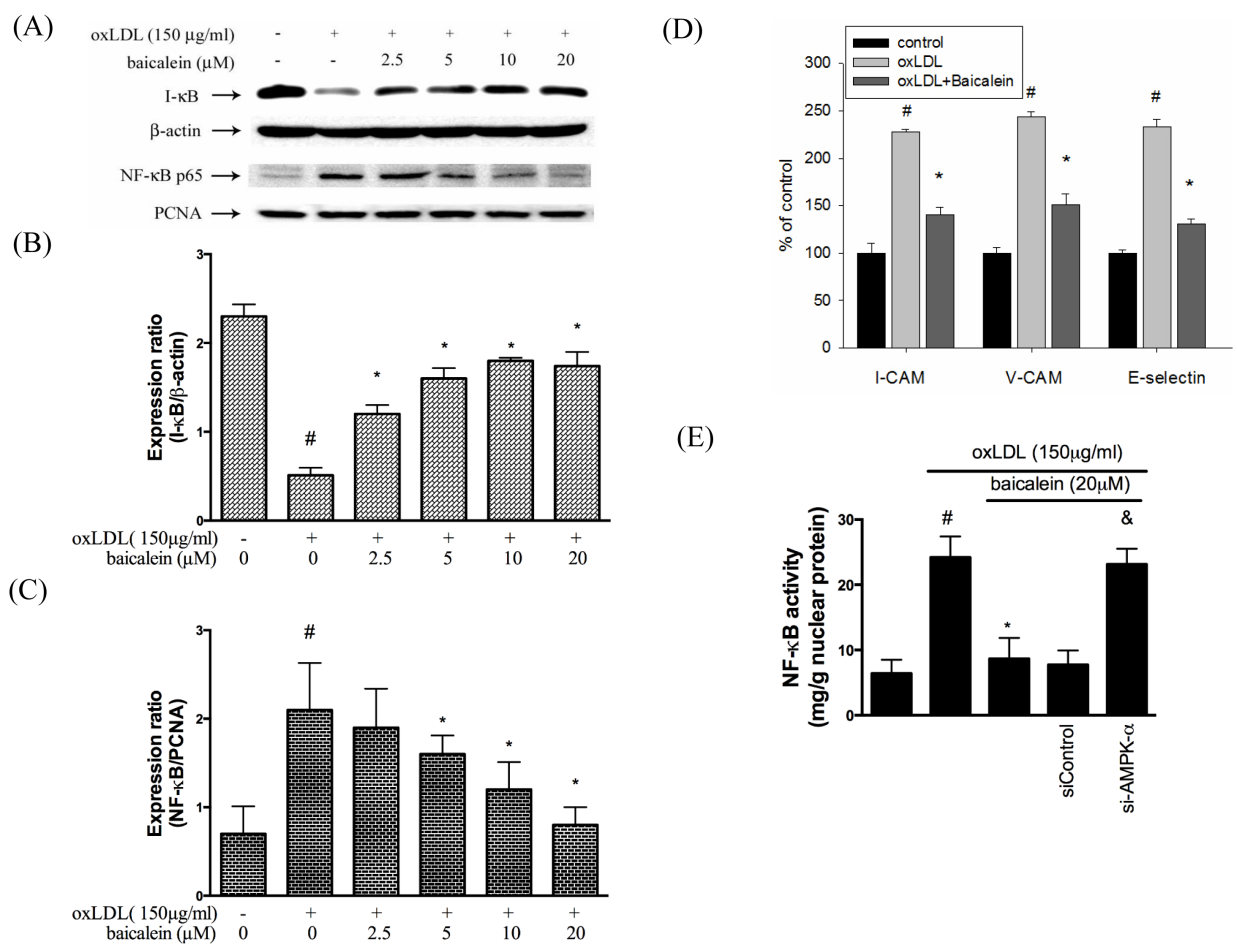
Effects of baicalein on oxLDL-induced NF-kB activation and adhesion molecular expression **A.**, **B.**, **C.** The protein expression of cytosolic IκB and nucleus NF-κB were analyzed by Western blot pretreated with baicalein (2.5-20 μM) for 2 hrs prior to exposure to oxLDL(150 μg/ml) in HUVECs. Anti-β-actin and anti-PCNA antibody were used for normalization of cytosolic and nuclear proteins, respectively. control and *P < 0.05 compared with oxLDL-stimulated HUVECs. **D.** The histogram of cell surface expression of VCAM-1, ICAM-1, and E-selectin was generated by flow cytometry. **E.** Nucleic proteins were extracted for nuclear translocation assay of NF-κBp65. Data are means±SEM of 3 independent analyses. # *P* < 0.05 compared with control, & *P* < 0.05 compared with baicalein 20 μM and *P < 0.05 compared with oxLDL-stimulated HUVECs.

As shown in Figure 7A-7C, NF-κBp65 protein expression levels were up-regulated and I-κB protein expression levels were down-regulated in oxLDL-treated endothelial nuclear fraction. However, this finding is restored by baicalein intervention. Next, we investigated whether baicalein represses NF-κB-triggered downstream inflammatory proteins. As shown in Figure 7D, the expression of E-selectin, VCAM, ICAM were up-regulated in endothelial cells that had been stimulated with oxLDL for than in the non-treated HUVECs but not in cells which were pretreated with 20 μM baicalein. As expected, Figure 7E shown that AMPK silencing impaired the ability of NF-kB reduction by baicalein.

## DISCUSSION

Oxidized low density lipoprotein (oxLDL) in circulation system is one of well-known risk factors in human cardiovascular diseases [6]. The integrating of oxLDL to LOX-1 stimulates NADPH oxidase activation and quick increase intracellular ROS generation. Meanwhile, ROS accumulation in endothelial cell up-regulates LOX-1 expression. This present study showed that baicalein could suppress endothelial LOX-1 protein expression due to suppressing ROS generation. Pretreatment with inhibitors of apocynin, PKC or anti-LOX-1 mAb prevents oxLDL-facilitated superoxide anion formation, suggesting that the integrating of oxLDL to LOX-1 and the consequent generation of red radical by NADPH oxidase via PKC may be the key procress in LOX-1 medicated endothelial injuries.

Vascular cells to oxidative stress is a operation of the equalization between the oxidative damage and the antioxidant defense capability [19]. Therefore, endothelial dysfunction caused by oxLDL resulted in ROS generation and antioxidant enzymes dysfunction, thereby induced endothelial death by triggering ROS-sensitive mechanism [20]. We found that baicalein intervention inhibited ROS formation and oxLDL-impaired SOD-1 levels (Figure 3). Therefore, we suggested that baicalein mitigates oxLDL-induced oxidative injuries could be through the increase the anti-oxidant enzymes activity and the capacity of ROS scavenging.

We assumed that the mechanisms by which baicalein inhibites oxLDL-caused endothelial dysfunction could be mainly through the ROS scavenger with SOD activity.

NADPH oxidase is the key origin of ROS generation in human vessel [21]. We found that intervention with a NADPH oxidase pharmacological inhibitor (apocynin) significantly suppressed oxLDL-induced ROS generation and baicalein also suppress oxLDL-induced NADPH oxidase protein expression and suppress the assembly of NADPH oxidase enzyme complex p47phox and Rac-1 membrane translocation. The present results showed that the pretreatment of HUVECs with baicalein abolished oxLDL-activated expression of NADPH oxidase and ROS production. A previous study showed high glucose causes ROS generation by NADPH oxidase is mediated by the DAG-PKC pathway in human endothelial cells. They also demonstrated that the inhibition of NADPH oxidase-generated oxidative stress require the up-regulation of AMPK because this effect is completely abolished by Compound C (AMPK inhibitor) and enhanced by AICAR (AMPK activator) [15]. And Li et al. suggested a mechanism for LOX-1 in oxLDL-caused MMP-1 and MMP-3 activation in endothelial cells are mediated by LOX-1 and PKC-β signaling. They suggest that LOX-1 induced NADPH oxidase activation is modulated by protein kinase C and NADPH oxidase-promoted endothelial dysfunction and MMPs expression is inhibited by AMPK [22]. Our study found that oxLDL can induce PKC-α and PKC-β activation and is mitigated by AMPK (Figure 5). Incubation with baicalein significantly increased AMPK phosphorylation after 48 hrs in HUVECs (Figure 4). Pretreated with baicalein suppressed oxLDL-induced PKC-α and PKC-β activation. The effects of baicalein suppressed PKC activation in oxLDL exposure cells are antagonized in the presence of Compound C or AMPK knockdown cells. Furthermore, pretreatment with AICAR reversed oxLDL-induced PKC activation (Figure 5). Thus, we strongly suggesting that baicalein attenuates oxLDL-induced protein kinase C and NADPH oxidase subunits p47phox and Rac-1 membrane translocation is repressed by AMPK activation.

In summary, the present results indicated that baicalein attenuates LOX-1-mediated endothelial oxidative dysfunction via modulating AMPK / PKC / NADPH oxidase / NF-κB signaling. Therefore, reduce the downstream of NADPH oxidase-caused ROS formation and dysfunction of SOD-1. Our report adds to an evidence that baicalein has positive effects on human cardiovascular diseases.

## MATERIALS AND METHODS

### Cell culture and reagents

Human umbilical vein endothelial cells (HUVECs) were obtained from ATCC. HUVECs were cultured with M199 basal medium supplemented with low-serum growth supplement and penicillin (50 IU/ml)-streptomycin (50 μg/ml). Trypsin-EDTA was used to passage cells. M199 and trypsin-EDTA were obtained from Gibco (Grand Island, NY, USA). Low-serum growth supplement was purchased from Cascade (Portland, OR, USA). Additionally. Baicalein, AICAR, Compound C, Apocynin, penicillin and streptomycin were all purchased from Sigma (St. Louis, MO, USA). Anti-β-actin, anti-AMPK, anti-AMPK-α, anti-phospho AKT, anti-LOX-1, anti-PKCα, anti-PKCβ, anti-PKCγ, anti-PKCδ, I-κB, NF-κBp65, anti-Cu, Zn SOD, anti-Mn SOD were all obtained from Santa Cruz Biotechnology (Santa Cruz, CA, USA).

### Lipoprotein separation

Human plasma was obtained from the Taichung Blood Bank (Taichung, Taiwan) and LDL was isolated using sequential ultracentrifugation (=1.019-1.063 g/ml) in KBr solution containing 30 mM EDTA, stored at 4°C in sterile, dark environment and used within 3 days as previously described. Immediately before the oxidation tests, LDL was separated from EDTA and from diffusible low molecular mass compounds by gel filtration on PD-10 Sephadex G-25 Mgel (Pharmacia) in 0.01 mol/l phosphate-buffered saline (136.9 mmol/l NaCl, 2.68 mmol/l KCl, 4 mmol/l Na2HPO4,1.76 mmol/l KH2PO4) at pH 7.4. Cu2+-modified LDL (1mg protein/ml) was prepared by exposing LDL to 10 μM CuSO4 for 16 hrs at 37°C. Protein concentration was determined by Bradford Protein Assay.

### Measurement of ROS production

The effect of baicalein on ROS production in HUVECs was determined by a fluorometric assay using 2′,7′-dichlorofluorescein acetoxymethyl ester (DCF-AM). Confluent HUVECs (10^4^ cells/well) in 96-well plates were preincubated with various concentrations of baicalein for 2 hrs; After the removal of medium from wells, cells were incubated with 10 μM DCF-AM for 1 hr. oxLDL was then added to the medium in the absence or presence of baicalein for 2 hrs. The fluorescence intensity was measured with a fluorescence microplate reader (Labsystem, CA) calibtated for exciation at 485 nm and emission at 538 nm. The percentage increase in fluorescence per well was calculated by the formula [(Ft2-Ft0)/Ft0] X 100, where Ft2 is the fluorescence at 2 hrs of oxLDL exposure and Ft0 is the fluorescence at 0 min of oxLDL exposure.

### Immunoblotting

To determine whether baicalein could ameliorate the oxLDL-induced protein. HUVECs were grown to confluence, pretreated with baicalein for 2 hrs and then stimulated with oxLDL for 24 hrs. At the end of stimulation, cells were washed, scraped from dishes, and lysed in RIPA buffer (in mM: HEPES 20, MgCl2 1.5, EDTA 2, EGTA 5, dithiothreitol 0.1, phenylmethylsulfonyl fluoride 0.1, pH 7.5). Proteins (30 μg) were separated by electrophoresis on SDS-polyacrylamide gel. After the protein had been transferred to polyvinylidene difluoride membrane (Millipore, Bedford, MA), the blots was incubated with blocking buffer (1X PBS and 5% nonfat dry milk) for 1 hr at room temperature and then probed with primary antibodies overnight at 4°C, followed by incubation with horseradish peroxidase-conjugated secondary antibody (1:5000) for 1 hr. To control equal loading of total protein in all lanes, blots were stained with mouse anti-β-actin antibody at a 1:50000 dilution. The bound immunoproteins were detected by an enhancer chemiluminescent assay (ECL; Amersham, Berkshire, UK). The intensities were quantified by densitometric analysis (Digital Protein DNA Imagineware, Huntington Station, NY).

### AMPK silencing

The day before the experiment, the cells were harvested by trypsinization, resuspended in complete medium at the concentration of 1×10^5^ cells/mL, and kept at 37°C, while the transfection complex was being prepared. Gene silencing of AMPK was performed by specific synthesized siRNA for AMPKα1 (validated siRNA, Santa Cruz, CA, USA). The day of the experiment, transfection siRNA (10 nM) or scrambled sequence as control was incubated with Polyplus Transfection Reagent (Qiagen, Hilden, Germany) following manufacturer's instructions. After 10 min incubation at room temperature, the obtained complexes were added drop-wise onto the cells sub-cultured in replaced culture medium. The cells were maintained in a 37°C incubator until analysis. After 24hrs, 48hrs and 72hrs from transfection, the cells were collected for protein and gene expression analyses of AMPK. Cell viability of all the samples exceeded 80%.

### Membrane protein extraction

We used Mem-PER^®^ Eukaryotic Membrane Protein Extraction Reagent Kit to extract membrane protein. Cells grown to 80% confluency and subjected to various treatments were subsequently washed with ice-cold PBS and it was prepared for membrane protein extraction. Cells grown on 10-cm dish gently scraped with 3 ml ice-cold PBS and it was centrifuged at 10,000x g for 10 min at 4°C. After carefully aspirating the supernatant, cells added 150 μl of Reagent A to the cell pellet. Pipette up and down to obtain a homogeneous cell suspension. Incubate 10 minutes at room temperature with occasional vortexing, then added 450 μl of diluted Reagent C to each tube of lysed cells and vortex. Incubate tubes on ice for 30 minutes, vortexing every 5 minutes. Centrifuge tubes at 10,000x g for 3 minutes at 4°C. Transfer supernatant to new tubes and incubate 10 minutes in 37°C water bath to separate the membrane protein fraction. Centrifuge tubes at room temperature for 2 minutes at 10,000 g to isolate the hydrophobic fraction (i.e., the fraction containing membrane protein of interest) from the hydrophilic fraction, Carefully remove the hydrophilic phase (top layer) from the hydrophobic protein phase (bottom layer) and save in a new tube. Perform phase separations as quickly as possible because the interface between the layers slowly disappears at room temperature. Place the separated fractions on ice. The supernatants (membrane protein extracts) were stored aliquots at −80°C. Protein concentration of the supernatants were determined by the colorimetric assay (Bradford).

### Nuclear protein extraction

Cells grown to 80% confluency and subjected to various treatments were subsequently washed with ice-cold PBS and it was prepared for nuclear protein extraction. Cells grown on 10-cm dish were gently scraped with 3 ml ice-cold PBS and it were centrifuged at 1,000x g for 10 min at 4°C. After carefully aspirating the supernatant, cells were resuspended with 200 μl ice-cold BUFFER-I (10 mM Hepes (pH 8.0), 1.5 mM MgCl2, 10 mM KCl, 1 mM dithiothreitol, and proteinase inhibitor cocktail (Roche Molecular Biochemicals) and incubated for 15 min on ice to allow cells to swell, followed by adding 20 ll IGEPAL-CA630. After vigorously vortexing for 10 s and centrifuging at 16,000 g for 5 min at 4°C, the supernatant (cytoplasmic fraction) were carefully aspirated and the pellet were resuspended with ice-cold BUFFER-II (20 mM Hepes (pH 8.0), 1.5 mM MgCl2, 25% glycerol, 420 mM NaCl, 0.2 mM EDTA, 1 mM dithiothreitol and proteinase inhibitor cocktail (Roche Molecular Biochemicals)) and vigorously vortex. After vortexing, the suspension was placed on ice for 30 min before centrifuging at 16,000x g for 15 min at 4°C. The supernatants (nuclear extracts) were stored aliquots at −80°C. Protein concentration of the supernatants was determined by the colorimetric assay (Bradford). NF-κB activity was measured by an NF-κB p65 Active ELISA kit (Imgenex, San Diego, CA) according to the manufacturer's instructions. The absorbance at 405 nm was determined using a microplate reader (spectraMAX 340).

### Adhesion molecule expression

To determine whether baicalein could modify the oxLDL-induced adhesion molecule expression, HUVECs were grown to confluence, pretreated with baicalein for 2 hrs and stimulated with oxLDL (150 μg/ml) for 24 hrs. At the end of stimulation, HUVECs were harvested and incubated with FITC-conjugated anti-ICAM-1, anti-VCAM-1 and anti-E-selectin (R&D, Minneapolis, MN) for 45 min at room temperature. After the HUVECs had been washed three times, their immunofluorescence intensity was analyzed by flow cytometry using a Becton Dickinson FACScan flow cytometer (Mountain View, CA, USA).

### Statistical analyses

All experiments were repeated 3 times, and one of these results is provided. Results are expressed as mean±SEM. Differences between the groups were analyzed using one-way ANOVA followed by the Student's t test. A P-value < 0.05 was considered statistically significant.
